# Predicting post-stroke functional outcome using explainable machine learning and integrated data

**DOI:** 10.1038/s41598-026-47814-x

**Published:** 2026-04-15

**Authors:** Jesper Olsson, Tara M. Stanne, Björn Andersson, Christina Jern

**Affiliations:** 1 Department of Laboratory Medicine, Institute of Biomedicine, Sahlgrenska Academy, University of Gothenburg, Box 440, 405 30 Gothenburg, Sweden; 2 Department of Clinical Genetics and Genomics, Sahlgrenska University Hospital, Gothenburg, Region Västra Götaland Sweden; 3 Bioinformatics and Data Centre, University of Gothenburg, Gothenburg, Sweden

**Keywords:** Biomarkers, Medical research, Neurology, Neuroscience

## Abstract

Functional outcome after acute ischemic stroke (AIS) varies widely, and existing prognostic scores may not capture complex relationships. We evaluated a diverse set of clinical characteristics and blood biomarkers with multiple machine learning models to predict 3-month functional outcome after AIS, and used explainable artificial intelligence to identify key drivers of performance. Models were trained on 506 patients aged 18–69 years with AIS enrolled at four stroke units in western Sweden. We compared extreme gradient boosting, multilayer perceptron (MLP), and L1- and L2-regularized logistic regression. Feature importance was assessed with Shapley additive global explanations. Of the 506 patients, 105 had an unfavorable outcome (modified Rankin Scale score > 2). All models showed high area under the curve (AUROC, 0.900–0.906). The MLP achieved the highest precision–recall performance (AUPRC, 0.773 ± 0.080) and sensitivity (0.655 ± 0.096), though with lower specificity (0.920 ± 0.035). Stroke severity (NIH Stroke Scale score) was the dominant predictor across models. Among biomarkers, brain-derived tau (BD-tau) was most informative, followed by inflammation-related plasma proteins. In conclusion, machine learning accurately predicted functional outcome after AIS. BD-tau and inflammation-related proteins contributed predictive information above stroke severity, suggesting a potential for blood biomarkers to enhance individualized prognostication after AIS.

**Supplementary Information:**

The online version contains supplementary material available at 10.1038/s41598-026-47814-x.

## Introduction

Ischemic stroke remains a leading cause of adult disability worldwide, and recent epidemiological data indicate a troubling rise in incidence among younger age groups^1^. Functional recovery after stroke varies widely across individuals, highlighting the need for more precise, individualized prognostic tools^2^. Traditional scores for functional outcome prediction, e.g. ASTRAL^3^, rely on a limited set of clinical predictors and assume linear relationships, which may fail to capture complex, nonlinear interactions inherent in biological systems.

Machine learning (ML) approaches offer a powerful alternative for modeling complex diseases and allow the use of integrated heterogeneous data sources to model intricate patterns that elude conventional statistical approaches. In various medical fields, ML methods such as random forest, gradient-boosted trees, and neural networks have outperformed standard prognostic indices^4–6^. Few studies have evaluated ML methods for predicting post-stroke functional outcome, but existing research has demonstrated some improvement in predictive ability over traditional models^7–10^. However, concerns over the “black-box” nature of the decision processes in these models introduce challenges for clinical adoption.

Explainable artificial intelligence (XAI) methods, such as those for computing feature-importance metrics, can increase transparency by revealing which inputs drive predictions and predictive performance. Some ML methods, such as random forest, allow for inherent estimation of feature importance due to its ensemble nature, while others, such as neural networks, do not allow for direct derivation of feature importance metrics^11,12^. This necessitates model-agnostic methods for feature importance. XAI methods in the context of feature importance can generally be divided into two categories: local and global^13^. Local explanation methods aim to measure which features contributed most to an individual prediction, while global explanation methods measure which features most affect overall model behavior across predictions.

Shapley additive explanations (SHAP) is a commonly used feature importance method that uses techniques from game theory to compute model-agnostic feature importance metrics^14^ and has previously been used to interpret predictions of post-stroke functional outcome, e.g.^15–18^. However, SHAP focuses on providing local explanations by measuring each feature’s impact on the model prediction (e.g. predicted probability) and only provides global explanations indirectly by aggregating feature impact from multiple predictions. It does not account for whether impactful features contributed positively or negatively to model *performance*. Shapley additive global explanations (SAGE), on the other hand, builds on the concepts of SHAP by using the underlying methods to provide global explanations that directly measure the contribution of each feature to model performance across a set of cases, such as a test set^19^.

Proteomic data have been successfully utilized in conjunction with ML methods to improve prognostic performance across a range of diseases. For instance, a recent study used proteomics and ML approaches to predict major adverse cardiovascular events^20^. We recently performed a proteomic profiling study and identified novel associations between inflammation-related proteins and functional outcome after acute ischemic stroke (AIS)^21^. Before this, multiple blood biomarkers have been shown to be associated with post-stroke functional outcome^22,23^, e.g.^24,25^. However, the few studies evaluating a combined ML and blood biomarkers approach for predicting post-stroke outcomes have only used a limited number of routine biomarkers and shown relatively poor performance^16–18,26,27^.

In this work, we leverage a richly annotated prospective cohort study on AIS of adults before 70 years of age to evaluate the potential of ML methods for predicting unfavorable 3-month functional outcome. The data consists of clinical characteristics and a broad set of blood biomarkers collected both within clinical routine and for research purposes, including proteomic measurements of inflammation-related proteins. Furthermore, we use XAI methods to assess feature importance and identify the features driving predictive performance.

## Methods

### Data source and study population

This study uses data from the Sahlgrenska Academy Study on Ischemic Stroke (SAHLSIS), an observational, longitudinal cohort study^28^. The study recruited 600 patients with AIS aged 18–69 years from four stroke units in western Sweden between 1998 and 2003. AIS was defined as patients rapidly developing clinical signs of focal disturbance of cerebral function lasting more than 24 h without hemorrhage or signs of another cause on neuroimaging. Maximum stroke severity within the first seven days of index stroke was scored using the Scandinavian Stroke Scale (SSS), and the SSS score was converted to a NIH Stroke Scale (NIHSS) score using an established algorithm^29^. The recruitment to the study took place before recanalization therapy was part of routine clinical treatment. While this limits the generalizability of the findings to modern stroke care, it provides an opportunity to model biological processes of stroke recovery in a treatment-naïve setting. Beyond collection of clinical and demographic data, as described elsewhere^28,30–32^, blood samples were biobanked.

The procedures for blood sampling, pre-analytical conditions, and biobanking have been described in detail elsewhere^33,34^. In brief, venous blood samples were drawn between 8:30 a.m. and 10:30 a.m. after overnight fast in the acute phase of ischemic stroke (median [interquartile range, IQR]^3–6^ days after index stroke). Plasma and serum were isolated within 2 h by centrifugation at 2000 × *g* at 4 °C for 20 min, aliquoted, and stored at − 80 °C pending analysis. Analyses of plasma/serum levels of a large number of proteins, e.g. hemostatic factors, inflammatory-related proteins, growth factors and brain injury biomarkers were performed as detailed in the supplementary methods. In addition, blood biomarkers analyzed within the clinical routine work-up at our hospital’s Department of Clinical Chemistry were also included in the data, and these analyses are also detailed in the supplementary methods. Assessments of baseline clinical characteristics were performed by clinicians blinded to the outcome, and analyses of blood biomarkers were performed by technicians blinded to clinical information.

The prespecified outcome was functional outcome after 3 months as assessed at an in-person visit by a stroke neurologist by the modified Rankin Scale (mRS). This outcome was dichotomized into favorable (mRS score 0–2) and unfavorable (mRS score 3–6) outcome.

### Ethical approval

Written informed consent was obtained by all participants or next-of-kin prior to enrollment. SAHLSIS was approved by the Regional Ethics Review Board in Gothenburg, Sweden (registration numbers L 653-97, 469-99, 469-99 T553-03, 413-04, 413-04 T586-13, and 413-04 T665-07) and performed in accordance with the 1964 Helsinki Declaration.

### Computational workflow

#### Data preprocessing

We excluded any cases with missing outcome (3-month mRS score), a recurrent stroke within 3 months or with > 20% missingness across features and removed features with > 30% missingness across cases. This resulted in a dataset consisting of 506 cases and 144 features. Missing values of binary and categorical features were imputed with their most common value. Categorical variables were one-hot encoded (i.e. using indicator variables with binary values 0 or 1 for each level). Numerical features were imputed by k-nearest neighbors (kNN) regression with k = 5. All variables were then standardized to a mean of zero and standard deviation of one. This was performed before kNN imputation for numerical features to prevent biased distance comparisons.

#### Machine learning workflow

We employed a robust evaluation strategy using repeated stratified five-fold cross-validation (CV) with ten repeats to estimate generalization performance. In each CV iteration, the data was randomly divided into five groups. This created a total of 50 modeling iterations, hereafter referred to as *fold iterations*. In each fold iteration, models were trained to predict unfavorable functional outcome on four training folds, i.e. 80% of the data, and evaluated on the held-out test fold, i.e. the remaining 20% of the data. Performance statistics were thus computed across a total of 50 test folds. Due to the repetition of CV, the test folds will not all be independent but overlapping. Prior to model training, the Boruta feature selection algorithm was applied to the training set to identify a stable subset of informative features^35^. During feature selection, Boruta iteratively trains a random forest model on an expanded dataset that includes the original features and *shadow features* (shuffled versions of the original features). The algorithm uses feature importance estimates to identify relevant features as those that significantly outperform the best-performing shadow feature.

Hyperparameter tuning was performed on each training set using a nested five-fold CV. Performance metrics used to evaluate the models included area under the receiver operating characteristic curve (AUROC), area under the precision-recall curve (AUPRC), F_1_ score, sensitivity, and specificity. Four ML methods were compared: ridge-regularized logistic regression, least absolute shrinkage and selection operator (LASSO), extreme gradient boosting (XGBoost), and a multilayer perceptron (MLP).

Logistic regression (LR) models the probability of a binary outcome by applying the logistic function to a weighted sum of input features. Regularization can be applied in the form of a penalty to the magnitude of the coefficients to reduce overfitting and improve generalization. Ridge LR uses an L_2_ penalty, which shrinks all coefficients toward zero proportionally, thereby stabilizing coefficient estimates while retaining the features. LASSO uses a stricter L_1_ penalty, which can shrink some coefficients exactly to zero^36^. In this study, the regularization hyperparameter was tuned using nested five-fold CV.

XGBoost is an optimized implementation of gradient-boosted decision trees, where trees are built sequentially to correct the errors of prior trees^37^. At each step, the model minimizes a loss function (e.g. logistic loss for classification) using gradient and second-order (Hessian) information, while applying regularization to control tree complexity. Multiple hyperparameters were tuned via a randomized search of 150 samples of the parameter space. Further details on the XGBoost optimization can be found in the supplementary methods.

A multilayer perceptron (MLP) is a feedforward neural network composed of an input layer, one or more hidden layers of fully connected units, and an output layer. Each unit computes a weighted sum of its inputs followed by an activation that usually performs a nonlinear transformation. The network’s parameters are learned by backpropagating the loss computed on the outputs, iteratively adjusting them to minimize the loss function using its gradient. Because of the nonlinearity introduced by the activation functions, the MLP transforms the feature space to unravel complex geometries and capture more intricate decision boundaries. This allows for the modeling of complex relationships, including interactions between features, that linear models cannot. In this study, the MLP was constructed with four hidden layers with 32, 64, 32, and 16 units, respectively, all using exponential linear unit (ELU) activation. No systematic hyperparameter tuning was performed on the MLP. Instead, the MLP architecture and hyperparameters were developed empirically on a separate CV of the data. Further details on the MLP can be found in the supplementary methods.

#### Feature importance

To derive model-agnostic global feature importance rankings, we computed SAGE^19^ values for each trained model and fold iteration. SAGE quantifies how much a model’s overall performance depends on each feature by computing the change in cross-entropy loss when that feature is withheld across all subsets of features. The values were computed on the test set of each iteration and averaged for each feature across all fold iterations. If a feature was rejected by Boruta during feature selection in a given fold iteration, but retained in at least one other, it was given a SAGE value of zero for that fold iteration. To allow for comparison of feature importance between models, the averages were then normalized. As such, the normalized mean SAGE values represent the mean estimated proportion of performance attributable to each feature.

Univariate associations between variables and outcome were assessed using the Wilcoxon rank-sum test for continuous variables and the Chi-squared test for categorical variables in R 4.5.2. The modeling was performed using Python 3.13. Scikit-learn 1.6.1 was the main package used for the modeling workflow, including its implementations for LASSO and Ridge logistic regression^38^. XGBoost 3.0.2 was used for XGBoost implementation and the MLP was developed and trained using PyTorch 2.7.1^39^. SAGE values were computed using sage-importance 0.0.6. Further details on the modeling workflow can be found in the supplementary methods.

### Code availability

Source code for this article is available in the following Github repository: https://github.com/jespeols/predicting_mRS_xML_integrated_data.

## Results

### Study population

After filtering cases with missing outcome, a recurrent stroke within 3 months, or too many missing features, the dataset consisted of 506 AIS patients aged 18–69 years from the SAHLSIS cohort. Demographic and clinical characteristics of this study population can be found in Table 1. The median age of patients was 58 (IQR: 52–64) and 63% were male. 105 of 506 patients (21%) had an unfavorable 3‑month outcome (mRS score 3–6), and most of these had moderate or moderately severe disability (mRS score distribution, score: no. (%): 0: 72 (14.2%); 1: 121 (23.9%); 2: 208 (41.1%); 3: 70 (13.8%); 4: 32 (6.3%); 5: 1 (0.2%); and 6: 2 (0.4%)). The median NIHSS score among all patients was 2.9 (IQR: 1.2–6.8), 2.0 (IQR: 0.7–3.8) in the favorable outcome group and 12.4 (IQR: 6.8–16.2) in the unfavorable outcome group. Characteristics tables containing comparisons between the two outcome groups for all clinical and blood biomarker variables can be found in the supplementary material (Supplementary Tables S1 and S5, respectively).

**Table 1:** Baseline clinical characteristics of the SAHLSIS cohort. Data are shown for patients in the whole cohort and those in the subgroups of favorable (mRS score 0–2) and unfavorable (mRS score 3–6) 3-month functional outcome after AIS. Baseline characteristics for the two outcome groups were compared and *p*-values < 0.05 were considered statistically significant and are indicated in bold.

Variable		3-month functional outcome		
	Overall N = 506	Favorable N = 401	Unfavorable N = 105	*p*-value
Age, years, median [IQR]	58 [52–64]	58 [52–64]	59 [53–65]	0.6
Sex, male, no. (%)	319 (63)	248 (62)	71 (68)	0.3
Hypertension, no. (%)	301 (60)	238 (59)	63 (61)	> 0.9
Diabetes mellitus, no. (%)	94 (19)	70 (17)	24 (23)	0.3
Hyperlipidemia, no. (%)	362 (75)	284 (74)	78 (81)	0.2
Atrial fibrillation, no. (%)	52 (11)	29 (7.8)	23 (25)	** < 0.001**
Coronary artery disease, no. (%)	85 (17)	64 (16)	21 (21)	0.3
Smoker, no. (%)	196 (39)	159 (40)	37 (36)	0.5
Body mass index [kg/m^2^], median [IQR]	25.9 [23.8–28.7]	26.0 [23.9–28.7]	25.5 [23.0–28.7]	0.3
Previous stroke, no. (%)	99 (20)	72 (18)	27 (26)	0.10
Stroke severity (NIHSS score), median [IQR]	2.9 [1.2–6.8]	2.0 [0.7–3.8]	12.4 [6.8–16.2]	** < 0.001**
TOAST, no. (%)				**0.001**
Large artery atherosclerosis	57 (11)	43 (11)	14 (13)	
Small artery occlusion	111 (22)	101 (25)	10 (9.5)	
Cardioembolic	84 (17)	61 (15)	23 (22)	
Cryptogenic	136 (27)	111 (28)	25 (24)	
Other determined	39 (7.7)	24 (6.0)	15 (14)	
Undetermined	79 (16)	61 (15)	18 (17)	
OCSP, no. (%)				** < 0.001**
LACI	189 (37)	166 (42)	23 (22)	
POCI	126 (25)	114 (29)	12 (11)	
TACI/PACI	190 (38)	120 (30)	70 (67)	

mRS, modified Rankin Scale; IQR, Interquartile range; BMI, Body mass index; NIHSS, National Institutes of Health Stroke Scale; TOAST, Trial of Org 10172 in Acute Stroke Treatment; OCSP, Oxford Community Stroke Project; LACI, lacunar cerebral infarct; POCI, posterior circulation infarct; TACI, total anterior circulation infarct; PACI, partial anterior circulation infarct.

### Model performance

Table 2 presents a performance comparison of the ML methods in the form of averaged metrics computed on the 50 test folds. The models achieve similar performance and variability across metrics. In area under the precision-recall curve (AUPRC), the MLP achieves the highest average performance at 0.773 ± 0.080, and the highest area under the receiver operating characteristic curve (AUROC) score at 0.906 ± 0.033. The MLP also achieves the highest sensitivity score at 0.655 ± 0.096, which is qualified by the lowest specificity score at 0.920 ± 0.035. In contrast, Ridge achieves the highest specificity at 0.957 ± 0.023, but lowest sensitivity at 0.553 ± 0.099. Notably, the MLP and XGBoost achieve higher F_1_ score (0.667 ± 0.075 and 0.671 ± 0.078, respectively) than Ridge and LASSO (0.642 ± 0.086 and 0.647 ± 0.080, respectively).

**Table 2:** Performance of ML methods predicting 3-month unfavorable functional outcome (mRS score 3–6) after ischemic stroke. Scores are expressed as mean ± standard deviation. Bold indicates the best-performing model for each metric.

Model	AUPRC	AUROC	F1	Sensitivity	Specificity
MLP	**0.773 ± 0.080**	**0.906 ± 0.033**	0.667 ± 0.075	**0.655 ± 0.096**	0.920 ± 0.035
LASSO	0.767 ± 0.077	0.902 ± 0.033	0.647 ± 0.080	0.572 ± 0.096	0.950 ± 0.025
Ridge	0.762 ± 0.079	0.900 ± 0.034	0.642 ± 0.086	0.553 ± 0.099	**0.957 ± 0.023**
XGBoost	0.756 ± 0.073	0.904 ± 0.030	**0.671 ± 0.078**	0.618 ± 0.108	0.944 ± 0.024

### Feature importance

**Fig. 1:**
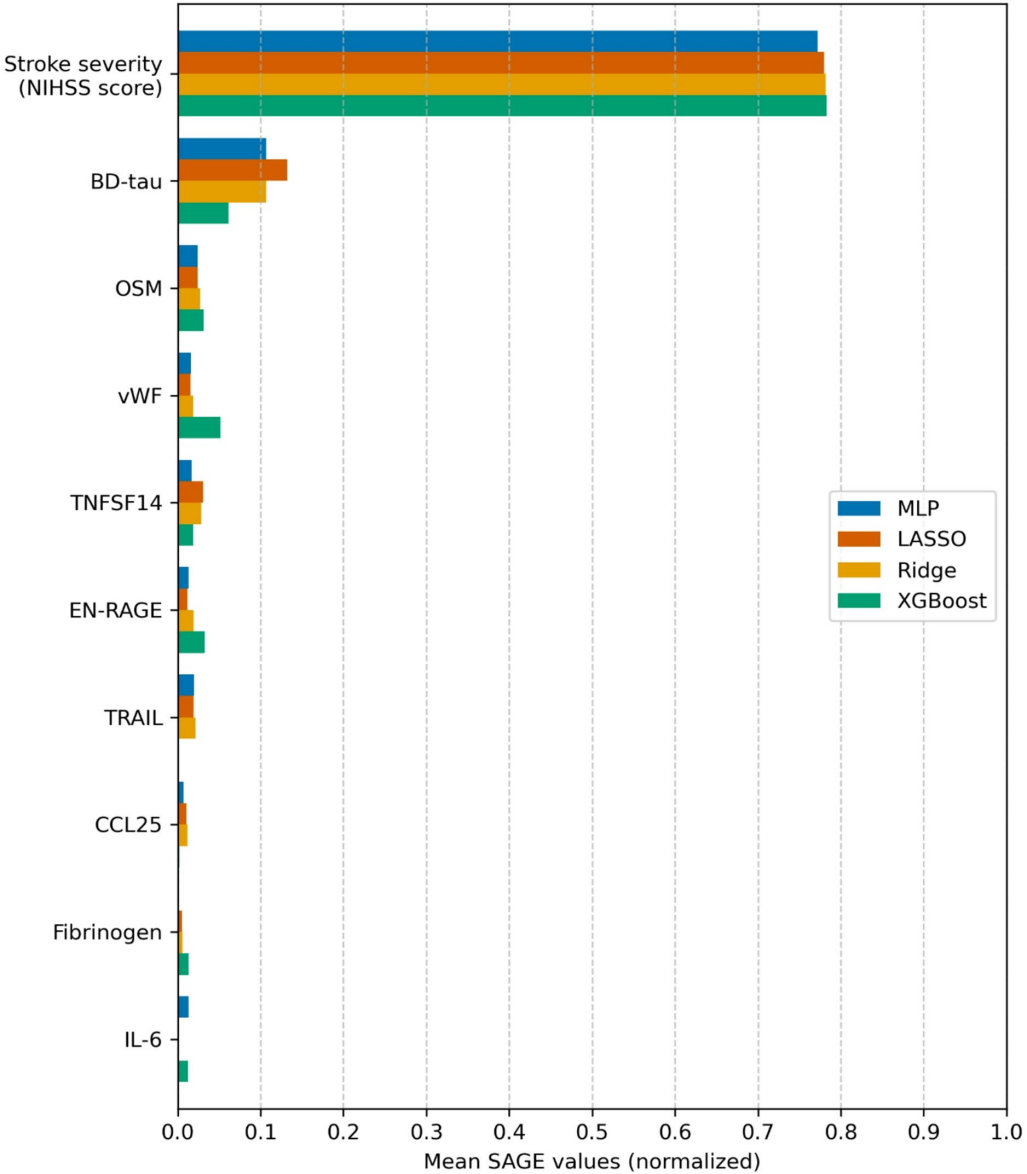
Normalized mean SAGE values computed across the 50 test folds. Top 10 features ranked by averaging across models are shown. NIHSS, NIH Stroke Scale score; BD-tau, brain-derived tau; OSM, oncostatin M; vWF, von Willebrand factor; TNFSF14, tumor necrosis factor superfamily 14; EN-RAGE, aka. S100 calcium binding protein A12 (S100A12); TRAIL, TNF-related apoptosis-inducing ligand; CCL25, C–C motif chemokine ligand 25; IL-6, interleukin 6; MLP, multilayer perceptron; LASSO, least absolute shrinkage operator; XGBoost, extreme gradient boosting.

Feature importance rankings based on means of SAGE values computed on the 50 test folds are shown after normalization for the top 10 features in Fig. 1. Stroke severity (NIHSS score) is the most important predictor across all models, followed by the neuronal injury biomarker brain-derived tau (BD-tau). Further predictors that show lower relative importance consist of multiple inflammation-related proteins such as oncostatin M (OSM), tumor necrosis factor superfamily 14 (TNFSF14), and coagulation factor von Willebrand factor (vWF). Results from Boruta feature selection are presented in the supplement (Supplementary Table S4) and show that a relatively small share of the features pass feature selection, but that a subset pass in all 50 fold iterations.

## Discussion

This study aimed to evaluate the performance of various ML models in predicting unfavorable 3-month functional outcome after ischemic stroke using a richly annotated dataset comprising a broad set of clinical characteristics and diverse blood biomarkers, including proteomic data on inflammation-related proteins. Additionally, we sought to identify key predictors driving model performance using the model-agnostic feature importance method SAGE. The models achieved comparable performance across performance metrics, though the MLP demonstrated notably better performance in classifying unfavorable outcomes in this imbalanced dataset at the cost of lower specificity. Feature importance analysis revealed that stroke severity was the most important predictor of 3-month functional outcome across all models, with brain-derived tau (BD-tau) standing out among the remaining features.

Compared to previous studies evaluating ML methods for predicting post-stroke functional outcome, the present study uses a uniquely well-characterized cohort of adult ischemic stroke patients before 70 years of age, integrating routine clinical data with broad molecular data. While comparing findings from modeling across different studies carries many caveats due to differences in modeling strategy and evaluation on different datasets from study populations with different characteristics, it can still be insightful to compare the findings and conclusions of different approaches.

For instance, one previous study evaluated a MLP, random forest and logistic regression on 2604 AIS patients that did not receive recanalization treatment and showed that the MLP outperformed other approaches at predicting 3-month functional outcome^9^. Furthermore, it showed that the NIHSS score was the most important of the 38 predictors comprising mostly clinical characteristics and a small set of routine blood biomarkers. Our study solidifies this conclusion by examining a broader set of models and predictors. Another study evaluated a variety of models using five-fold CV and found that they performed comparably when predicting 6-month functional outcome on 1735 AIS patients with mainly clinical characteristics data^8^. However, in that study, the pattern of higher sensitivity at the cost of lower specificity for the MLP in comparison to other models was not observed. A third study by Lee et al. (2023) compared three tree-based models (including XGBoost) on 3687 AIS patients and used SHAP for feature importance to infer global explanations in terms of which of the 16 features (clinical characteristics and routine blood biomarkers) most impacted predictions^15^. In line with the present results, they found that initial stroke severity was the most important predictor. The other main contributing variables were age, white blood cell count (WBC), and early neurological deterioration (END). In our dataset, WBC was not selected among the most important features, and a likely explanation for this is that the present dataset included circulating levels of several circulating pro-inflammatory proteins that were not included in the study by Lee et al. We did not register data on END, which precludes comparisons on that specific variable.

The study by Lee et al. further found that common co-morbidities such as atrial fibrillation, diabetes mellitus, BMI, and hypertension had a negligible impact on model output, a finding that aligns with our results showing that these clinical variables did not pass feature selection. In all of these studies, age was reported as an important variable, while in the present study it does not pass feature selection even once. This is likely due to the restricted age range of 18–69 in the SAHLSIS cohort, while the previous studies had no such age limit. Indeed, the SHAP analysis in Lee et al. showed that after 75 years of age, the impact of the age variable on model output (i.e. predicted probability of unfavorable outcome) increased notably^15^.

One previous study used a modeling approach similar to ours, comparing among others MLP, XGBoost, and LR using Boruta feature selection on a variable set containing routine clinical variables and blood biomarkers for 470 ischemic or hemorrhagic stroke patients^40^. Importantly, however, the outcome was dichotomized mRS at discharge. That said, they found that the MLP slightly outperformed XGBoost and LASSO, and observed the same pattern of improved prediction of unfavorable outcomes at the cost of lower specificity as in the present study. Using SHAP, they identified NIHSS score upon admission as the dominant predictor. However, they only applied SHAP to the best-performing model, while our study adds robustness to that conclusion by comparing relative feature importance across models.

Further examination of the performance results in Table 2 reveals a key trade-off between the different models. Due to the class imbalance of the dataset, there are fewer examples of unfavorable outcomes for the models to learn from, and predicting cases of unfavorable outcome will generally be more difficult for the models than predicting favorable ones. It is therefore interesting that the more complex models, namely MLP and XGBoost, were better at predicting unfavorable outcomes and balancing the two classes at the standard decision cutoff of 0.5, as shown by their higher F_1_ scores. As we look at performance across cutoffs via AUPRC and AUROC, the picture changes and the models converge. However, the results indicate that there is some predictive information contained within non-linearity in the predictors and/or interactions between them. Therefore, more complex models may be more useful for data-driven research into the underlying mechanisms of stroke recovery, especially when combined with XAI methods that alleviate their black-box nature. Furthermore, it is likely that these models will scale better with more data. However, for implementation of data-driven methods in clinical practice, the transparency and simplicity of linear models carry notable benefits. On the other hand, these results indicate that such a model would require a tuned cutoff to avoid missing a substantial share of unfavorable outcomes.

Feature importance analysis using SAGE showed that stroke severity (captured in this study as the maximum NIHSS score within seven days of index stroke) is the most important predictor of 3-month functional outcome across all models. This aligns with the well-established central role of neurological deficit in post-stroke prognostication^41^. The particularly dominant contribution observed in our cohort likely reflects both the intrinsic relationship between stroke severity and outcome, and the fact that our severity measure captures the peak neurological deficit rather than a single time-point assessment (e.g. admission or 24 h post-stroke) which may underestimate the true maximal deficit. Given that most prior studies rely on NIHSS score measured at a single time point, the predictive capability of stroke severity may be comparatively increased in our analysis and likely contributes to the strong relative importance of stroke severity observed in this cohort. Notably, however, BD-tau emerged as the most important blood biomarker predictor, suggesting it provides prognostic information beyond what is captured by the NIHSS score. We have previously investigated the role of BD-tau as a biomarker for post-stroke outcomes, and found an association between BD-tau and functional outcome after ischemic stroke^42^ that has been reported in other cohorts, as well^43^. Furthermore, we have previously investigated how BD-tau and the NIHSS score perform as predictors of outcome depending on infarct location and hypothesized that including BD-tau may alleviate the bias toward anterior circulation stroke present in the NIHSS score^44,45^.

Beyond the NIHSS score and BD-tau, a number of inflammation-related proteins show up with similar performance contribution. Among these, OSM, TNFSF14, EN-RAGE (aka. S100A12), CCL25, interleukin-6 (IL-6) and tumor necrosis factor-related apoptosis-inducing ligand (TRAIL) show up among the most important. This finding concords with a previous investigation we conducted into associations between inflammation-related proteins and 3-month functional outcome on the same cohort^21^. In this study, we extend this analysis by applying a more comprehensive ML methodology on a broader data set of multiple data types. The present results suggest that proteomics data may provide prognostic information in AIS, but that the NIHSS score remains the main predictor of post-stroke functional outcome. Further research including proteomics data in larger cohorts may help clarify the potential of these biomarkers in prognostication. Furthermore, broader protein panels with coverage beyond inflammation-related proteins may provide a wider biological context and more information on the pathophysiological processes underlying stroke recovery.

The primary strength of this study is the extensive data on the patients in the SAHLSIS cohort, including thorough clinical workup and laboratory analysis of different types of blood biomarkers, including among others hemostatic factors, inflammation-related proteins and markers of immune system activity and neuronal injury. Beyond the extensive annotation, there are additional strengths of the cohort. First, due to the organization of the Swedish medical system, the cohort offers good representation of stroke patients within the given age range and time period within the geographical region of Västra Götaland and does not suffer from selection bias from which hospital patients arrive to. Second, the age restriction of the cohort carries with it the benefit of fewer co-morbidities. Third, this study employs a comprehensive modeling workflow consisting of preprocessing, feature selection, and nuanced evaluation with multiple performance metrics of a diverse set of models of varying complexity. Furthermore, the use of XAI to rigorously analyze and fairly compare feature importance across models allows for robust identification of drivers of unfavorable functional outcome.

There are important limitations of this study that need to be considered. First, since no independent validation set was used, the results and conclusions of the study are limited to the analyzed cohort. This study focused on ischemic stroke at working age, which limits the generalizability of the results to older patients. Furthermore, as inclusion was performed in a time era when recanalization therapies were not yet part of standard routine care, the results cannot be generalized to patients receiving these treatments. Second, while the data is comprehensive, it does not include data on history of mental illness or pre-stroke cognitive function, mRS pre-stroke or at discharge, or information on post-stroke rehabilitation. Further, imaging data was only available for a subset of this cohort (n = 254) and therefore not included in the analysis. That said, we hypothesize that much of the predictive information that can be gained from imaging is captured by other features such as NIHSS score and BD-tau. For instance, we have previously demonstrated strong correlation between BD-tau and infarct volume in the subgroup of this cohort with imaging^44^. Third, the relatively small sample size carries a risk of overfitting for complex models such as the MLP and XGBoost. However, by using Boruta to include only features deemed relevant to prediction, the models were fitted on much smaller feature sets, thereby reducing this risk. Fourth, while our study uses XAI methods to interpret models, we limit this to global explanations. Local explanations are relevant both in clinical contexts and for research, for example by investigating causes of misclassification or why different models make different decisions. Future research ought to combine SAGE with SHAP to explore what drives both performance and individual predictions. Finally, while this study explores the potential of combining blood biomarkers with machine‑learning approaches to support individualized outcome prediction, these findings are preliminary. Larger, cohorts will be required to validate these results, enable stratified analyses across clinically relevant subgroups, and determine whether such approaches can ultimately yield prognostic insights of tangible value for clinical decision‑making and future clinical trial design.

In conclusion, machine learning models that utilize integrated clinical data with blood-based biomarkers perform well at predicting 3-month functional outcome after ischemic stroke in the form of dichotomized mRS. All models achieved comparable performance, with the MLP slightly outperforming others in terms of AUPRC by better classification of unfavorable outcomes. Feature importance analyses using SAGE highlighted stroke severity and BD-tau as key predictors of unfavorable outcome. These findings emphasize the central role of acute stroke severity in shaping recovery trajectories after stroke, but indicate that including blood biomarkers in predictive models using ML methods holds promise in improving personalized outcome predictions. As the present study included ischemic stroke patients of working age who did not receive recanalization therapy, future studies applying and evaluating this modeling approach on larger and more diverse cohorts are warranted.

## Supplementary Information

Below is the link to the electronic supplementary material.

Supplementary Material 1

## Data Availability

Anonymized data will be shared upon reasonable request, provided data transfer agrees with EU legislation on the general data protection regulation and with decisions by the Ethical Review Board of Sweden and the University of Gothenburg, the latter which should be regulated in a data transfer agreement.
