# Supplementary material for: Predicting post-stroke functional outcome using explainable machine learning and integrated data
Source: Sci Rep. 2026 Apr 15;16:12462. doi: 10.1038/s41598-026-47814-x (PMC13083973; doi:10.1038/s41598-026-47814-x)
Supplement: Supplementary file 1 — Supplementary Material 1 [file 41598_2026_47814_MOESM1_ESM.pdf]

Supplementary Material

Predicting Post-Stroke Functional Outcome Using Explainable Machine Learning and Integrated Data

Supplementary methods

Clinical and demographic data

In this study we included the main clinical variables that we collected in SAHLSIS, which had a focus on vascular risk factors [1]. Examinations for determining these clinical variables were performed in the acute phase (1-10 days after index stroke) and/or at the 3-month follow-up visit, as described in more detail elsewhere [1]. In brief, the protocol included both questionnaires and examinations providing data on personal history of stroke and coronary artery disease (CAD), family history of stroke and myocardial infarction (MI), socioeconomic factors, self-perceived psychological stress [2,3], self-reported physical activity [3,4], smoking habits, alcohol consumption, as well as measurements of blood pressure and anthropometric variables such as waist and hip circumference. All clinical variables are characterized and compared between the two outcome groups in Supplementary Table S1.

Many of the clinical and demographic variables were categorical and consisted of multiple levels corresponding to different reply alternatives in the questionnaire. However, since the variables had several levels with very few cases, we dichotomized these to reduce the risk of overfitting. This may, however, decrease the prognostic value of the variable. Smoking was coded as current versus never or former (smoking cessation within the last year from inclusion). Alcohol consumption was categorized as alcohol consumption ≥4 times a week versus <4 times a week or never, based on self-assessment at inclusion. Occupational class was first coded into five categories according to the Swedish socioeconomic classification system [5] and then dichotomized into categories corresponding to lower and higher education [1]. History of coronary artery disease was defined as having suffered myocardial infarction or having ECG changes indicating previous myocardial infarction [1]. Living alone at time of index stroke was categorized as living alone versus living with a partner or and adult family member (sibling or child over the age of 20 years or a parent) [6]. Self-reported physical activity during leisure was coded as sedentary versus moderate, regular or tough exercise. Finally, high self-perceived psychological stress was coded as permanent stress for ≥1 year versus several or occasional stress periods in the last five years or having never experienced stress.

Hypertension was defined as pharmacological treatment for hypertension and/or systolic blood pressure ≥160 mmHg, and/or diastolic blood pressure ≥90 mmHg. Diabetes mellitus was defined as dietary or pharmacological treatment and/or fasting plasma glucose ≥7.0 mmol/L. Hyperlipidemia was defined as pharmacological treatment, total fasting serum cholesterol >5.0 mmol/L, and/or low-density lipoprotein (LDL) >3.0 mmol/L. All the measurements were performed at the 3-month follow-up.

We also collected data on personal history of several other diseases such as neurological, psychiatric, and autoimmune diseases. As few study participants were diagnosed with any of

these diseases, data on these comorbidities were not included in the present study. As an example, only one study participant was diagnosed with early-onset Alzheimer's disease and 5 with other neurological diseases as described elsewhere [7].

Index stroke severity was scored using the Scandinavian Stroke scale, which has been demonstrated to have comparable performance to the NIH Stroke Scale (NIHSS) in predicting death or dependence after stroke [8]. The score reflecting the highest neurological impairment within 7 days of hospital admission was converted to the NIHSS score using an established algorithm [9].

Etiological subtypes were classified according to the Trial of Org 10172 in Acute Stroke Treatment (TOAST) criteria [10]. The main subtypes were large artery atherosclerosis (LAA), small artery occlusion (SAO), and cardioembolic stroke (CE). Cryptogenic stroke was defined as when no cause was determined despite an extensive evaluation, while undetermined stroke was used for when more than one cause was found or when evaluation was insufficient. Furthermore, other determined stroke was used for arterial dissections and other minor causes. Stroke cases were also classified according to the Oxford Community Stroke Project (OCSP) criteria [11,12].

Blood biomarker analyses

A subset of the blood biomarkers in this data were analyzed in accordance with standard protocols during clinical routine at the Department of Clinical Chemistry at the Sahlgrenska University Hospital in Gothenburg, Sweden. These include total and differential leukocyte counts, thrombocyte count, erythrocyte volume fraction, total cholesterol, low- and high-density lipoprotein, triglycerides, sodium, potassium, hemoglobin, prothrombin, aspartate aminotransferase, alanine transaminase, alkaline phosphatase, activated partial thromboplastin, bilirubin, homocystein, uric acid, vitamin B12, s- and b-folic acids, creatinine, insulin and plasma glucose. Estimated glomerular filtration rate (eGFR) was calculated using the revised Lund-Malmö Study equation [13].

Most blood biomarkers in this data were, however, analysed outside clinical routine. These are detailed in Supplementary Table S2. For each analyte, the same batch of reagents were used. Plasma proteomics analyses were performed using the Olink Proseek Multiplex Inflammation panel I [14] (Olink Bioscience, Uppsala, Sweden), and our methodology has been described in detail elsewhere [15]. 65 of the 92 proteins met a >80% call-rate and were transformed to $\log_2$ normalized protein expression (NPX) units, with values below the limit of detection (LOD) imputed with the value LOD/2. All blood biomarker variables are characterized and compared between the two outcome groups in Supplementary Table S5.

Boruta feature selection

The Boruta algorithm, implemented using the BorutaPy package in Python, was utilized for all-relevant feature selection [16]. The algorithm was executed for a maximum of 100 iterations. Features that remained in the tentative state (neither confirmed nor rejected) after the process were retained for subsequent analysis. To expedite the evaluation, the BorutaPy implementation's feature of automatic tree count selection was employed, which reduces the number of trees as features are progressively rejected.

Multilayer perceptron

The multilayer perceptron (MLP) was implemented and trained using the PyTorch 2.7.1 package [17] in Python. The MLP architecture and its hyperparameters were developed empirically on a separate five-fold cross-validation (CV) of the data. The MLP architecture consists of an input layer, four hidden layers, and an output layer. The input layer projects the features into a 32-dimensional space (i.e. 32 artificial neurons, or units, in the layer), followed by an exponential linear unit (ELU) activation function. The subsequent hidden layers apply batch normalization before ELU activation. The first three hidden layers apply dropout with a probability of 0.5 (i.e. setting half of the weights to zero) after activation. The dimensionality is expanded to 64 units in the second layer, then reduced to 32 and 16 units in the following layers before the final output layer produces a one-dimensional logit (unnormalized output).

To train the model, the AdamW optimizer [18] was used to minimize the binary cross-entropy loss. AdamW was configured with a learning rate of 10^{-3} and weight decay coefficient of 10^{-4}. A batch size of 32 was used for the training, and the model was run for 100 epochs. However, to prevent overfitting, the training was monitored by evaluating the loss on a validation set composed of a randomly selected 10% of the training set. If the validation loss did not improve for 7 subsequent epochs, the training was stopped early and the best-performing model was saved and used for evaluation on the test fold.

Hyperparameter optimization

Hyperparameter optimization of the XGBoost and logistic regression models was carried out in each fold iteration using nested five-fold CV that evaluated the binary cross-entropy loss (aka. negative log-likelihood loss). The search space of the regularization parameter of the logistic regression models included 100 evenly spaced values between 10^{-4} and 10^{4}. XGBoost hyperparameter optimization employed a randomized searjch approach where a specified number of randomly selected points (here 150) in the search space are evaluated. The XGBoost hyperparameters tuned and their associated search spaces are presented in Supplementary Table S3.

Feature importance

Global feature importance was assessed using Shapley additive global explanations (SAGE) [19] and implemented using the sage-importance 0.0.6 package in Python. SAGE values were computed on the test set of each fold iteration and the train set was used to learn the conditional distributions of the features. The conditional feature distributions were estimated using their marginal distributions (the built-in MarginalImputer function). Then, the SAGE values were computed using permutation sampling (PermutationEstimator). These methods are described in the original paper [19].

Supplementary Tables

Supplementary Table S1: Characteristics of clinical variables in the analysis, characterized on the SAHLSIS cohort. Baseline characteristics for the favorable (mRS score 0-2) and unfavorable (mRS score 3-6) outcome groups were compared.

Variable	Overall N = 506	3-month functional outcome Favorable N = 401	Unfavorable N = 105
Age, years, median [IQR]	58 [52-64]	58 [52-64]	59 [53-65]
Sex, male, no. (%)	319 (63)	248 (62)	71 (68)
Hypertension, no. (%)	301 (60)	238 (59)	63 (61)
Diabetes mellitus, no. (%)	94 (19)	70 (17)	24 (23)
Hyperlipidemia, no. (%)	362 (75)	284 (74)	78 (81)
Atrial fibrillation, no. (%)	52 (11)	29 (7.8)	23 (25)
Coronary artery disease, no. (%)	85 (17)	64 (16)	21 (21)
Smoker, no. (%)	196 (39)	159 (40)	37 (36)
Alcohol consumption, >4 times/week, no. (%)	38 (8.0)	28 (7.4)	10 (10)
Snus user, no. (%)	53 (12)	42 (12)	11 (12)
Body mass index [kg/m²], median [IQR]	25.9 [23.8-28.7]	26.0 [23.9-28.7]	25.5 [23.0-28.7]
Previous stroke, no. (%)	99 (20)	72 (18)	27 (26)
Family history of stroke, no. (%)	201 (41)	170 (43)	31 (31)
Family history of myocardial infarction, no. (%)	187 (38)	145 (37)	42 (42)
Stroke severity (NIHSS score), median [IQR]	2.9 [1.2-6.8]	2.0 [0.7-3.8]	12.4 [6.8-16.2]
TOAST, no. (%)			
Cryptogenic	136 (27)	111 (28)	25 (24)
Small artery occlusion	111 (22)	101 (25)	10 (9.5)
Cardioembolic	84 (17)	61 (15)	23 (22)
Undetermined	79 (16)	61 (15)	18 (17)
Large artery atherosclerosis	57 (11)	43 (11)	14 (13)
Other determined	39 (7.7)	24 (6.0)	15 (14)
OCSP, no. (%)			
TACI/PACI	190 (38)	120 (30)	70 (67)
LACI	189 (37)	166 (42)	23 (22)
POCI	126 (25)	114 (29)	12 (11)
Sagittal diameter [cm], median [IQR]	18.5 [16.0-21.0]	18.5 [16.5-21.3]	18.0 [16.0-20.0]
Waist-hip ratio, median [IQR]	0.95 [0.90-0.99]	0.95 [0.90-0.99]	0.95 [0.92-0.99]

Systolic blood pressure [mmHg], median [IQR]	146 [130-165]	146 [130-165]	148 [129-165]
Diastolic blood pressure [mmHg], median [IQR]	80 [75-90]	80 [75-90]	85 [75-93]
Living alone, no. (%)	142 (29)	108 (27)	34 (33)
Self-reported leisure physical activity, sedentary, no. (%)	87 (18)	59 (16)	28 (29)
Self-perceived psychological stress, high, no. (%)	101 (21)	76 (20)	25 (27)
Employment status, unemployed/retired, no. (%)	225 (46)	171 (44)	54 (53)
Occupation, lower education, no. (%)	301 (62)	232 (60)	69 (66)
Warfarin treatment, no. (%)	50 (9.9)	38 (9.5)	12 (11)
Time until sampling [days], median [IQR]	4 [3-6]	4 [3-6]	4 [2-6]

Abbreviation: mRS, modified Rankin Scale; IQR, Interquartile range; BMI, Body mass index; NIHSS, National Institutes of Health Stroke Scale; TOAST, Trial of Org 10172 in Acute Stroke Treatment; OCSP, Oxford Community Stroke Project; LACI, lacunar cerebral infarct; POCI, posterior circulation infarct; TACI, total anterior circulation infarct; PACI, partial anterior circulation infarct.

Supplementary Table S2: Description of assays used for blood biomarkers not analyzed in clinical routine. References are to publications in which the present data was first published (if applicable), or where the assay was used by others.

Biomarker(s)	Method	Serum/plasma	Citation
C-reactive protein, insulin	Solid-phase chemiluminescent immunometric assay (IMMULITE 2000, Diagnostic Product Corporation, USA)	Serum	[20,21]
HOMA-IR	insulin x glucose	Serum	[22]
von Willebrand factor	ELISA kit (Asserachrom VWF:Ag, Diagnostica Stago, Asnières, France)	Citrate plasma	[23]
tPA antigen	ELISA kit (TintElize® t-PA, Biopool International, Umeå, Sweden)	Citrate plasma	[24]
tPA activity	Biofunctional immunosorbant assay (BIA, Chromolize™ tPA, Biopool International	Citrate plasma	[24]
PAI-1 antigen	ELISA kit (COALIZA® PAI-1 Chromogenix, Haemochrom Diagnostica AB, Mölndal, Sweden)	Citrate plasma	[24]
Fibrinogen	Automated clot rate assay	Citrate plasma	[25]
TAFI	In-house ELISA	Citrate plasma	[26]
TAFI released active peptide activity	In-house ELISA	Citrate plasma	[26]
TAFIai	In-house TAFI activity assay	Citrate plasma	[27]
FSAP antigen	In-house ELISA	Citrate plasma	[28]
FSAP activity	In-house FSAP activity assay	Citrate plasma	[28]
FVII antigen	ELISA kit (Total Human FVIIag Assay; Molecular Innovations, Novi, MI, USA)	Citrate plasma	[29]
TAT antigen	ELISA kit (IMUBIND TAT, American Diagnostica Inc., Greenwich, CT)	Citrate plasma	[30]
Erythropoetin, VEGF	Human Hypoxia Serum/Plasma Assay, MSD® 96-Well MULTI-SPOT® (Meso Scale Discovery, Rockville, Maryland, USA)	Serum	[31,32]
IGFBP1	ELISA kit (IGFBP1 kit, Mediagnost, Reutlingen, Germany)	Serum	[33]
GCSF, FGF2	ELISA kit (Quantikine HS, #HSFB00D; R&D Systems, Minneapolis, MN)	Serum	[34]

IGF2	ELISA kit (IGF2 kit, Mediagnost, Reutlingen, Germany)	Serum	[35]
BDNF	ELISA kit (BDNF Emax ImmunoAssay System, Promega, Madison, WI)	Serum	[36]
RANTES	ELISA kit (Human RANTES, Thermo Scientific)	Serum	[37]
BD-tau	SiMoA Homebrew (Quanterix, MA, USA)	EDTA plasma	[7]
P-tau217	SiMoA Homebrew (Quanterix, MA, USA)	EDTA plasma	[38]
65 inflammation-related proteins	Olink ProSeek Multiplex Inflammation panel I (Olink Bioscience, Uppsala, Sweden)	EDTA plasma	[15]

Abbreviations: HOMA-IR, Homeostatic Model Assessment of Insulin Resistance; ELISA, Enzyme-linked immunosorbent assay; tPA, Tissue-type plasminogen activator; PAI-1, Plasminogen activator inhibitor-1; TAFI, Thrombin-activatable fibrinolysis inhibitor; TAFIai, activated thrombin-activatable fibrinolysis inhibitor; FSAP, Factor VII–activating protease; FVII, Factor VII; TAT, Thrombin-antithrombin complex; VEGF, Vascular endothelial growth factor; IGFBP1, Insulin-like growth factor-binding protein 1; GCSF, Granulocyte colony-stimulating factor; FGF2, Fibroblast growth factor 2; IGF2, Insulin-like growth factor 2; BDNF, Brain-derived neurotrophic factor; BD-tau, Brain-derived tau; Ptau217, Phosphorylated tau 217.

Supplementary Table S3: Hyperparameters optimized for XGBoost using five-fold cross-validation and their associated search spaces.

Parameter	Description	Search space
`n_estimators`	Number of boosting rounds (final number of trees)	[100, 200, 300, 500]
`max_depth`	Maximum depth of each decision tree	[3, 5, 7]
`learning_rate`	Shrinkage controlling the contribution of each tree	[0.05, 0.1, 0.2, 0.3]
`subsample`	Fraction of training samples used per tree	[0.8, 0.9, 1.0]
`colsample_bytree`	Fraction of features sampled for each tree	[0.8, 0.9, 1.0]
`reg_lambda`	L_2 regularization term on weights	[0, 0.5, 1.0, 5.0, 10.0]
`min_child_weight`	Minimum sum of instance weights required in a child node	[1, 3, 5, 7, 10]

Supplementary Table S4: Number of folds in which a feature was selected by Boruta along with their type. Showing features that were selected in at least one of the 50 total fold iterations. A colon (:) in the feature name indicates that the feature is a binary indicator variable derived from a categorical variable with multiple levels, where the feature name is followed by the level it represents.

Feature	Folds selected (n=50)
Brain-derived tau	50
High-sensitivity C-reactive protein	50
EN-RAGE	50
IL-6	50
Stroke severity (NIHSS score)	50
Fibrinogen	49
von Willebrand factor	49
TNFSF14	44
TRAIL	42
OSM	40
CCL25	33
MCP3	18
Flt3L	8
uPA	7
HGF	6
IFNgamma	6
Alkaline phosphatase (ALP)	5
CCL23	3
Neutrophil-to-lymphocyte ratio	3
Insulin-like growth factor 2	2
OCSP: TACI/PACI	2
TRANCE	2
4EBP1	1
CSF1	1
CXCL10	1
Neutrophil count	1

Supplementary Table S5: Characteristics of blood biomarker variables in the analysis, measured on the SAHLSIS cohort. Baseline characteristics for the favorable (mRS score 0-2) and unfavorable (mRS score 3-6) outcome groups were compared.

Variable	Overall N = 506	3-month functional outcome Favorable N = 401	Unfavorable N = 105
**Measured by routine blood chemistry, median (IQR)**			
Creatinine [µmol/L]	93 [82-104]	93 [82-104]	94 [83-104]
Estimated glomerular filtration rate [mL/min/1,73 m²]	67 [60-74]	67 [60-75]	67 [60-72]
Plasma glucose [mmol/L]	5.60 [5.11-6.60]	5.55 [5.10-6.42]	6.00 [5.40-7.80]
Total leukocyte count [10^9/L]	8.00 [6.50-9.60]	8.00 [6.45-9.60]	8.00 [6.80-9.80]
Neutrophil count [10^9/L]	4.30 [3.40-5.60]	4.20 [3.20-5.20]	5.20 [3.90-6.60]
Lymphocyte count [10^9/L]	2.00 [1.50-2.50]	2.00 [1.50-2.50]	1.80 [1.40-2.30]
Neutrophil-to-lymphocyte ratio	2.23 [1.64-3.18]	2.06 [1.57-2.90]	2.90 [1.97-4.14]
Monocyte count [10^9/L]	0.50 [0.40-0.70]	0.50 [0.40-0.60]	0.60 [0.48-0.70]
Erythrocyte volume fraction [percentage]	43.0 [40.6-46.0]	43.0 [40.8-46.0]	43.2 [40.0-46.0]
Thrombocyte count [10/L]	255 [216-305]	257 [216-308]	249 [212-296]
Hemoglobin [g/L]	146 [136-155]	147 [137-155]	146 [136-156]
Cholesterol [mmol/L]	5.30 [4.60-6.20]	5.30 [4.60-6.20]	5.20 [4.40-6.00]
High-density lipoprotein [mmol/L]	1.20 [0.99-1.50]	1.20 [1.00-1.50]	1.12 [0.94-1.40]
Low-density lipoprotein [mmol/L]	3.30 [2.67-4.00]	3.30 [2.70-4.00]	3.20 [2.40-3.80]
Triglyceride [mmol/L]	1.40 [1.10-1.90]	1.40 [1.08-1.90]	1.40 [1.10-1.90]
Prothrombin [international normalized ratio]	1.00 [0.98-1.10]	1.00 [0.94-1.10]	1.00 [1.00-1.10]
Activated partial thromboplastin time [seconds]	31.0 [29.0-34.0]	32.0 [29.0-34.0]	31.0 [29.0-34.0]
Sodium [mol/L]	0.140 [0.138-0.141]	0.140 [0.138-0.141]	0.140 [0.138-0.141]
Potassium [mmol/L]	4.00 [3.80-4.30]	4.10 [3.80-4.30]	4.00 [3.80-4.30]
Aspartate aminotransferase (ASAT) [µkat/L]	0.43 [0.35-0.53]	0.43 [0.35-0.52]	0.44 [0.37-0.57]
Alanine transaminase (ALAT) [µkat/L]	0.44 [0.32-0.62]	0.44 [0.32-0.60]	0.45 [0.33-0.64]
Alkaline phosphatase (ALP) [µkat/L]	3.00 [2.50-3.70]	3.00 [2.53-3.70]	3.00 [2.40-3.75]
Bilirubin [µmol/L]	9.7 [7.0-13.0]	9.6 [7.0-12.0]	10.0 [7.2-14.0]
Homocysteine [µmol/L]	12.6 [10.6-15.5]	12.5 [10.5-15.4]	13.2 [10.9-16.6]
Uric acid/Urate [mmol/L]	0.31 [0.27-0.37]	0.31 [0.27-0.38]	0.31 [0.27-0.37]
B12 [nmol/L]	0.36 [0.28-0.45]	0.35 [0.27-0.45]	0.36 [0.29-0.54]

S-folic acid [nmol/L]	14 [9-20]	14 [10-20]	12 [8-20]
P-folic acid [µmol/L]	320 [220-420]	313 [210-420]	330 [240-436]
**Measured by single assays, median (IQR)**			
Insulin [µU/L]	11 [8-18]	11 [8-17]	13 [9-19]
HOMA-IR	2.56 [1.72-4.42]	2.53 [1.66-4.32]	2.69 [1.97-5.07]
High-sensitivity C-reactive protein [mg/L]	3 [1-9]	3 [1-6]	10 [4-27]
von Willebrand factor [IU/dl]	132 [99-168]	126 [96-160]	161 [128-202]
Tissue-type plasminogen activator (tPA) antigen [µg/L]	11.9 [8.8-15.7]	11.7 [8.6-15.3]	13.5 [10.3-17.7]
Tissue-type plasminogen activator (tPA) activity [µg/L]	0.63 [0.37-0.99]	0.59 [0.36-0.96]	0.77 [0.49-1.14]
Plasminogen activator inhibitor-1 antigen [µg/L]	53 [35-74]	53 [35-75]	51 [34-70]
Fibrinogen [g/L]	3.70 [3.05-4.31]	3.57 [2.98-4.11]	4.34 [3.44-5.12]
Thrombin-activatable fibrinolysis inhibitor (TAFI) [rel. to human plasma (%)]	94 [81-112]	94 [80-111]	96 [82-115]
TAFI released active peptide activity [rel. to human plasma (%)]	120 [104-138]	120 [104-138]	121 [104-138]
activated thrombin-activatable fibrinolysis inhibitor (TAFIai) [rel. to human plasma (%)]	104 [87-123]	104 [88-121]	103 [85-125]
Factor VII activating protease antigen (FSAP) [mg/L]	13.3 [11.2-16.2]	13.3 [11.3-16.1]	13.3 [11.0-16.2]
FSAP activity [U/mL]	1.27 [1.07-1.50]	1.27 [1.08-1.49]	1.29 [1.02-1.54]
Factor VII antigen [mg/L]	0.31 [0.24-0.38]	0.31 [0.24-0.38]	0.32 [0.25-0.38]
Erythropoietin [mIU/mL]	7.6 [5.8-10.6]	7.4 [5.7-10.3]	8.7 [6.7-12.0]
Vascular endothelial growth factor [ng/mL]	0.35 [0.18-0.60]	0.35 [0.19-0.58]	0.38 [0.16-0.70]
Insulin-like growth factor-binding protein 1 [µg/mL]	4 [2-8]	5 [2-8]	3 [1-8]
Insulin-like growth factor 2 [µg/mL]	0.73 [0.63-0.83]	0.74 [0.65-0.83]	0.66 [0.57-0.80]
Granulocyte colony-stimulating factor [pg/mL]	26 [19-36]	25 [18-36]	29 [22-46]
Brain-derived neurotrophic factor [µg/mL]	23 [16-29]	23 [16-29]	22 [15-27]
RANTES (CCL5) [pg/mL]	42 [28-61]	43 [29-61]	40 [22-59]
Brain-derived tau [pg/mL]	5 [4-15]	5 [4-10]	16 [6-39]
Phosphorylated tau 217 (p-tau217) [pg/mL]	0.51 [0.32-0.76]	0.50 [0.31-0.73]	0.61 [0.37-0.86]

Measured by Olink ProSeek Multiplex Inflammation I panel, NPX value, median (IQR)			
IL-8	6.18 [5.75-6.64]	6.09 [5.69-6.57]	6.46 [6.06-6.90]
VEGFA	10.70 [10.42-10.98]	10.66 [10.40-10.94]	10.81 [10.56-11.07]
MCP3	2.17 [1.76-2.58]	2.08 [1.63-2.48]	2.45 [2.19-2.91]
hGDNF	2.68 [2.48-2.97]	2.66 [2.46-2.94]	2.78 [2.55-3.07]
CDCP1	3.51 [3.12-3.93]	3.44 [3.12-3.88]	3.66 [3.19-4.08]
CD244	6.04 [5.82-6.28]	6.04 [5.82-6.27]	6.04 [5.78-6.29]
IL-7	2.31 [1.92-2.85]	2.28 [1.87-2.84]	2.50 [2.10-2.92]
OPG	10.26 [10.00-10.57]	10.25 [9.97-10.55]	10.33 [10.08-10.67]
LAPTGFbeta1	6.82 [6.54-7.17]	6.79 [6.50-7.14]	6.93 [6.73-7.22]
uPA	10.37 [10.17-10.60]	10.39 [10.19-10.62]	10.29 [10.07-10.51]
IL-6	3.23 [2.72-3.93]	3.05 [2.64-3.71]	3.93 [3.28-4.88]
MCP1	10.13 [9.85-10.45]	10.12 [9.85-10.43]	10.17 [9.87-10.58]
CXCL11	6.99 [6.41-7.62]	6.95 [6.41-7.62]	7.13 [6.50-7.65]
AXIN1	2.76 [2.04-3.85]	2.72 [2.02-3.62]	3.01 [2.28-4.31]
TRAIL	8.75 [8.52-9.01]	8.79 [8.59-9.05]	8.58 [8.24-8.88]
CXCL9	7.17 [6.68-7.76]	7.17 [6.69-7.77]	7.19 [6.66-7.67]
CST5	6.38 [6.11-6.74]	6.38 [6.12-6.73]	6.37 [6.00-6.77]
OSM	3.35 [2.63-4.11]	3.22 [2.53-3.94]	3.94 [3.17-4.84]
CXCL1	7.72 [6.77-8.60]	7.69 [6.62-8.60]	7.97 [7.25-8.54]
CCL4	5.55 [5.21-6.00]	5.52 [5.18-5.95]	5.62 [5.39-6.11]
CD6	3.43 [3.14-3.80]	3.45 [3.16-3.82]	3.29 [2.96-3.68]
SCF	7.91 [7.58-8.19]	7.93 [7.63-8.22]	7.82 [7.36-8.08]
IL-18	7.91 [7.52-8.27]	7.92 [7.52-8.26]	7.87 [7.53-8.32]
SLAMF1	2.56 [2.28-2.93]	2.54 [2.26-2.88]	2.61 [2.34-3.05]
MCP4	2.72 [2.30-3.15]	2.72 [2.30-3.16]	2.72 [2.30-3.09]
CCL11	7.62 [7.31-8.07]	7.62 [7.31-8.10]	7.65 [7.25-7.91]
TNFSF14	1.89 [1.60-2.23]	1.85 [1.52-2.14]	2.22 [1.84-2.61]
FGF23	2.67 [2.32-3.04]	2.68 [2.36-3.04]	2.62 [2.22-3.06]
LIFR	2.76 [2.51-3.00]	2.76 [2.51-2.98]	2.76 [2.54-3.05]
FGF21	5.58 [4.81-6.54]	5.58 [4.78-6.46]	5.55 [4.96-6.72]
CCL19	9.44 [8.93-10.03]	9.44 [8.94-10.01]	9.46 [8.93-10.10]
IL-10RB	6.27 [6.03-6.57]	6.26 [6.02-6.56]	6.31 [6.10-6.63]
IL-18R1	6.90 [6.57-7.21]	6.87 [6.54-7.18]	6.99 [6.69-7.33]
CXCL5	9.84 [8.29-10.96]	9.83 [8.21-	9.89 [8.61-

		11.00]	10.70]
TRANCE	4.58 [4.08-5.00]	4.66 [4.15-5.04]	4.32 [3.83-4.67]
HGF	7.01 [6.70-7.44]	6.96 [6.63-7.32]	7.35 [6.97-7.66]
IL-12B	4.14 [3.70-4.59]	4.18 [3.70-4.59]	4.06 [3.65-4.52]
MMP10	6.40 [5.97-6.89]	6.42 [5.99-6.89]	6.32 [5.90-6.79]
IL-10	3.17 [2.91-3.57]	3.13 [2.86-3.52]	3.37 [3.07-3.83]
CCL23	10.06 [9.78-10.39]	10.04 [9.75-10.34]	10.25 [9.94-10.60]
CD5	4.06 [3.83-4.36]	4.09 [3.86-4.38]	4.02 [3.72-4.29]
CCL3	2.69 [2.38-3.03]	2.65 [2.36-3.01]	2.79 [2.48-3.11]
Flt3L	9.02 [8.68-9.32]	9.06 [8.75-9.37]	8.81 [8.45-9.19]
CXCL6	6.66 [6.18-7.21]	6.65 [6.18-7.18]	6.73 [6.25-7.30]
CXCL10	8.90 [8.34-9.49]	8.79 [8.33-9.35]	9.25 [8.55-9.84]
4EBP1	5.67 [5.03-6.89]	5.53 [4.97-6.73]	6.18 [5.37-7.27]
SIRT2	3.68 [2.99-5.16]	3.59 [2.93-4.90]	4.12 [3.42-5.76]
CCL28	1.41 [1.19-1.72]	1.40 [1.19-1.69]	1.48 [1.20-1.81]
DNER	7.36 [7.14-7.58]	7.39 [7.19-7.60]	7.24 [7.02-7.46]
EN-RAGE	1.96 [1.53-2.53]	1.85 [1.47-2.32]	2.59 [1.94-3.11]
CD40	9.08 [8.81-9.39]	9.07 [8.80-9.37]	9.19 [8.90-9.48]
IFNgamma	1.11 [1.03-1.44]	1.13 [1.03-1.44]	1.04 [0.95-1.40]
FGF19	7.90 [7.30-8.58]	7.83 [7.25-8.54]	8.01 [7.51-8.67]
MCP2	8.59 [8.15-8.97]	8.60 [8.15-8.97]	8.56 [8.17-8.93]
CASP8	1.52 [1.24-1.94]	1.49 [1.22-1.85]	1.72 [1.36-2.29]
CCL25	6.19 [5.74-6.67]	6.25 [5.86-6.70]	5.90 [5.43-6.45]
CX3CL1	6.07 [5.77-6.34]	6.04 [5.78-6.32]	6.08 [5.76-6.39]
TNFRSF9	6.54 [6.24-6.89]	6.56 [6.27-6.91]	6.49 [6.13-6.79]
NT3	2.76 [2.49-3.08]	2.75 [2.49-3.07]	2.81 [2.49-3.10]
TWEAK	9.12 [8.83-9.36]	9.13 [8.85-9.39]	9.06 [8.72-9.25]
CCL20	6.40 [5.86-6.99]	6.35 [5.80-6.92]	6.65 [6.14-7.29]
STAMPB	4.00 [3.56-5.07]	3.94 [3.54-4.83]	4.45 [3.80-5.41]
ADA	5.29 [5.02-5.62]	5.28 [4.98-5.62]	5.30 [5.10-5.63]
TNFB	3.50 [3.19-3.82]	3.52 [3.24-3.83]	3.31 [3.06-3.65]
CSF1	7.97 [7.76-8.23]	7.93 [7.73-8.18]	8.13 [7.92-8.38]

Abbreviation: IQR, interquartile range; For full names of proteins measured by Olink ProSeek Multiplex Inflammation I panel, see [15] or the Olink website.

Supplementary References

1. Jood, K., Ladenvall, C., Rosengren, A., Blomstrand, C. & Jern, C. Family History in Ischemic Stroke Before 70 Years of Age. *Stroke* **36**, 1383–1387 (2005).

2. Rosengren, A., Tibblin, G. & Wilhelmsen, L. Self-perceived psychological stress and incidence of coronary artery disease in middle-aged men. *Am J Cardiol* **68**, 1171–1175 (1991).

3. Jood, K., Redfors, P., Rosengren, A., Blomstrand, C. & Jern, C. Self-perceived psychological stress and ischemic stroke: a case-control study. *BMC Medicine* **7**, 53 (2009).

4. Grimby, G. *et al.* The 'Saltin-Grimby Physical Activity Level Scale' and its application to health research. *Scand J Med Sci Sports* **25 Suppl 4**, 119–125 (2015).

5. Rosengren, A., Wedel, H. & Wilhelmsen, L. Coronary heart disease and mortality in middle aged men from different occupational classes in Sweden. *BMJ* **297**, 1497–1500 (1988).

6. Redfors, P. *et al.* Living alone predicts mortality in patients with ischemic stroke before 70 years of age: a long-term prospective follow-up study. *BMC Neurology* **16**, 80 (2016).

7. Stanne, T. M. *et al.* Association of Plasma Brain-Derived Tau With Functional Outcome After Ischemic Stroke. *Neurology* **102**, e209129 (2024).

8. Askim, T., Bernhardt, J., Churilov, L. & Indredavik, B. The Scandinavian Stroke Scale is equally as good as The National Institutes of Health Stroke Scale in identifying 3-month outcome. *J Rehabil Med* **48**, 909–912 (2016).

9. Gray, L. J., Ali, M., Lyden, P. D. & Bath, P. M. W. Interconversion of the National Institutes of Health Stroke Scale and Scandinavian Stroke Scale in Acute Stroke. *Journal of Stroke and Cerebrovascular Diseases* **18**, 466–468 (2009).

10. Harold P. Adams *et al.* Classification of subtype of acute ischemic stroke. Definitions for use in a multicenter clinical trial. TOAST. Trial of Org 10172 in Acute Stroke Treatment. *Stroke* **24**, 35–41 (1993).

11. de Andrade, J. B. C. *et al.* Oxfordshire Community Stroke Project Classification: A proposed automated algorithm. *Eur Stroke J* **6**, 160–167 (2021).

12. Bamford, J., Sandercock, P., Dennis, M., Burn, J. & Warlow, C. Classification and natural history of clinically identifiable subtypes of cerebral infarction. *Lancet* **337**, 1521–1526 (1991).

13. Björk, J., Grubb, A., Sterner, G. & Nyman, U. Revised equations for estimating glomerular filtration rate based on the Lund-Malmö Study cohort. *Scand J Clin Lab Invest* **71**, 232–239 (2011).

14. Assarsson, E. *et al.* Homogenous 96-Plex PEA Immunoassay Exhibiting High Sensitivity, Specificity, and Excellent Scalability. *PLOS ONE* **9**, e95192 (2014).

15. Angerfors, A. *et al.* Proteomic profiling identifies novel inflammation-related plasma proteins associated with ischemic stroke outcome. *Journal of Neuroinflammation* **20**, 224 (2023).

16. Kursa, M. B. & Rudnicki, W. R. Feature Selection with the Boruta Package. *Journal of Statistical Software* **36**, 1–13 (2010).

17. Paszke, A. *et al.* PyTorch: An Imperative Style, High-Performance Deep Learning Library. Preprint at https://doi.org/10.48550/arXiv.1912.01703 (2019).

18. Loshchilov, I. & Hutter, F. Decoupled Weight Decay Regularization. Preprint at https://doi.org/10.48550/arXiv.1711.05101 (2019).

19. Covert, I., Lundberg, S. & Lee, S.-I. Understanding Global Feature Contributions With Additive Importance Measures. Preprint at https://doi.org/10.48550/arXiv.2004.00668 (2020).

20. Matthews, D. R. *et al.* Homeostasis model assessment: insulin resistance and β-cell function from fasting plasma glucose and insulin concentrations in man. *Diabetologia* **28**, 412–419 (1985).

21. Claes Ladenvall *et al.* Serum C-Reactive Protein Concentration and Genotype in Relation to Ischemic Stroke Subtype. *Stroke* **37**, 2018–2023 (2006).

22. Åberg, D. *et al.* Homeostasis model assessment of insulin resistance and outcome of ischemic stroke in non-diabetic patients - a prospective observational study. *BMC Neurol* **19**, 177 (2019).

23. Hanson, E. *et al.* Plasma levels of von Willebrand factor in the etiologic subtypes of ischemic stroke. *J Thromb Haemost* **9**, 275–281 (2011).

24. Jood, K. *et al.* Fibrinolytic gene polymorphism and ischemic stroke. *Stroke* **36**, 2077–2081 (2005).

25. Jood, K., Danielson, J., Ladenvall, C., Blomstrand, C. & Jern, C. Fibrinogen gene variation and ischemic stroke. *J Thromb Haemost* **6**, 897–904 (2008).

26. Ladenvall, C. *et al.* Thrombin activatable fibrinolysis inhibitor activation peptide shows association with all major subtypes of ischemic stroke and with TAFI gene variation. *Arterioscler Thromb Vasc Biol* **27**, 955–962 (2007).

27. Ceresa, E. *et al.* Development of ELISAs measuring the extent of TAFI activation. *Arterioscler Thromb Vasc Biol* **26**, 423–428 (2006).

28. Hanson, E. *et al.* Plasma factor VII-activating protease antigen levels and activity are increased in ischemic stroke. *J Thromb Haemost* **10**, 848–856 (2012).

29. Stanne, T. M. *et al.* Factor VII antigen levels are differentially associated to etiological subtypes of ischaemic stroke. *Thromb Haemost* **110**, 1305–1306 (2013).

30. Połaska, P. *et al.* Coagulation abnormalities as predictors of renal dysfunction in heart failure with reduced ejection fraction. *Medical Studies/Studia Medyczne* **39**, 148–158 (2023).

31. Åberg, N. D. *et al.* Serum erythropoietin and outcome after ischaemic stroke: a prospective study. *BMJ Open* **6**, e009827 (2016).

32. Åberg, N. D. *et al.* Circulating levels of vascular endothelial growth factor and post-stroke long-term functional outcome. *Acta Neurol Scand* **141**, 405–414 (2020).

33. Åberg, D. *et al.* Serum IGFBP-1 Concentration as a Predictor of Outcome after Ischemic Stroke-A Prospective Observational Study. *Int J Mol Sci* **24**, 9120 (2023).

34. Wall, A. *et al.* Circulating granulocyte colony-stimulating factor and functional outcome after ischemic stroke: an observational study. *Neurol Res* **43**, 1013–1022 (2021).

35. Åberg, D. *et al.* Insulin-Like Growth Factor-II and Ischemic Stroke-A Prospective Observational Study. *Life (Basel)* **11**, 499 (2021).

36. Stanne, T. M. *et al.* Low Circulating Acute Brain-Derived Neurotrophic Factor Levels Are Associated With Poor Long-Term Functional Outcome After Ischemic Stroke. *Stroke* **47**, 1943–1945 (2016).

37. Chiang, J. J. *et al.* Viral unmasking of cellular 5S rRNA pseudogene transcripts induces RIG-I mediated immunity. *Nat Immunol* **19**, 53–62 (2018).

38. Gonzalez-Ortiz, F. *et al.* A novel ultrasensitive assay for plasma p-tau217: Performance in individuals with subjective cognitive decline and early Alzheimer's disease. *Alzheimers Dement* **20**, 1239–1249 (2024).
